# Supplementary material for: Type III Druantia two-component antiphage defense depends on the DruH-DruE interaction for inhibiting viral DNA circularization and replication
Source: Cell Insight. 2026 Jun 11;5(4):100341. doi: 10.1016/j.cellin.2026.100341 (PMC13316683; doi:10.1016/j.cellin.2026.100341)
Supplement: Multimedia component 1 [file mmc1.pdf]

Supplemental information

Type III Druantia two-component antiphage defense depends on the DruH-DruE interaction for halting viral DNA circularization and replication

Yakun Li, Zheng-Guo He*

State Key Laboratory of Virology and biosafety, Taikang Center for Life and Medical Sciences, TaiKang Medical School (School of Basic Medical Sciences), Wuhan University, Wuhan, 430071, China

*To whom correspondence should be addressed: School of Basic Medical Sciences, Wuhan University, Wuhan, 430071, China.
Email: hezhengguo2024@whu.edu.cn or hezhengguo2019@163.com

Tel: +86-27-68759222, Fax: +86-27-68759222

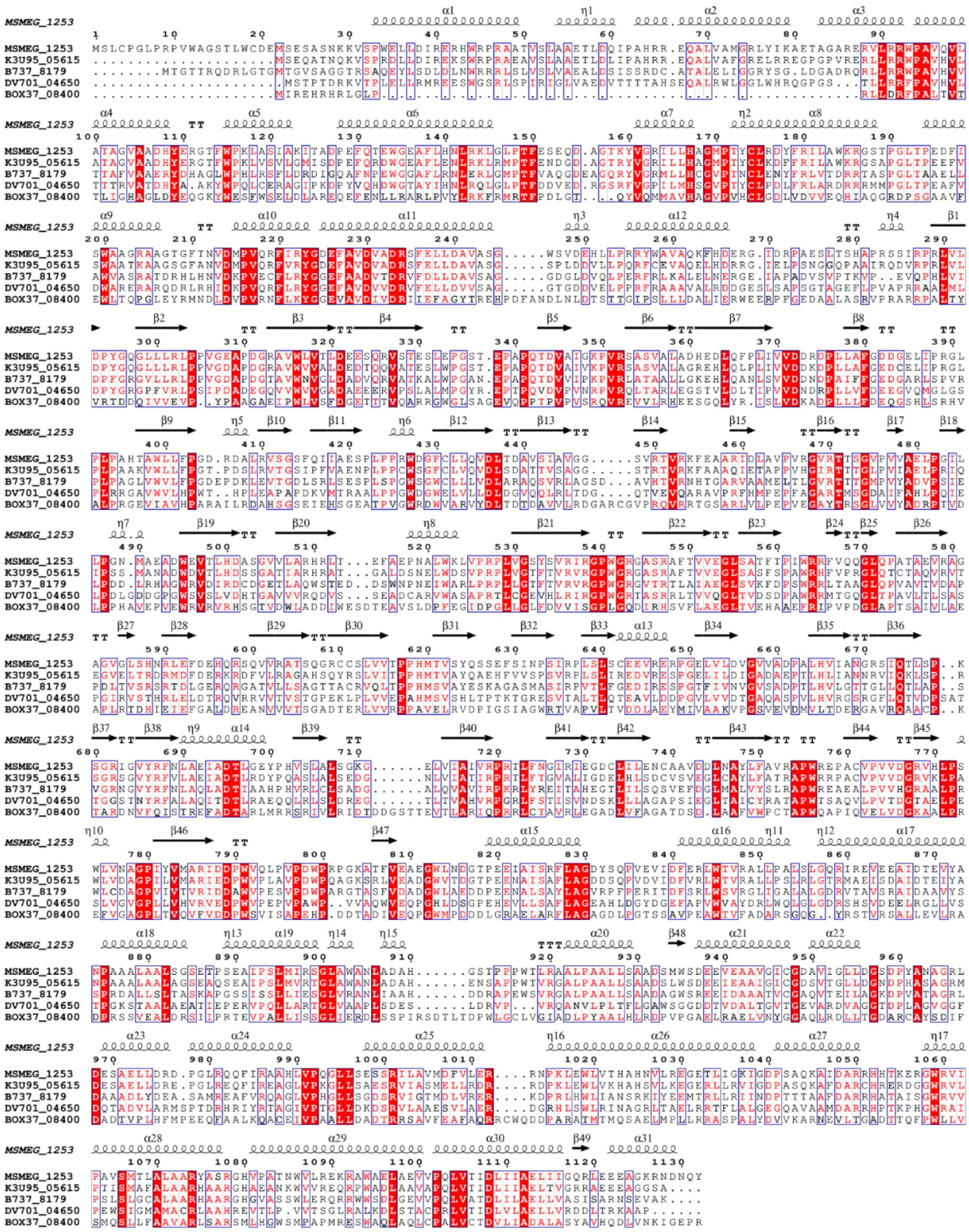

Fig. S1, related to Fig. 1. Amino acid sequence conservation analysis of DruH in the Type III Druantia system. The conservation of amino acids in DruH, a key component of the type III Druantia system, was analyzed and visualized using a color to represent the degree of conservation across homologous sequences.

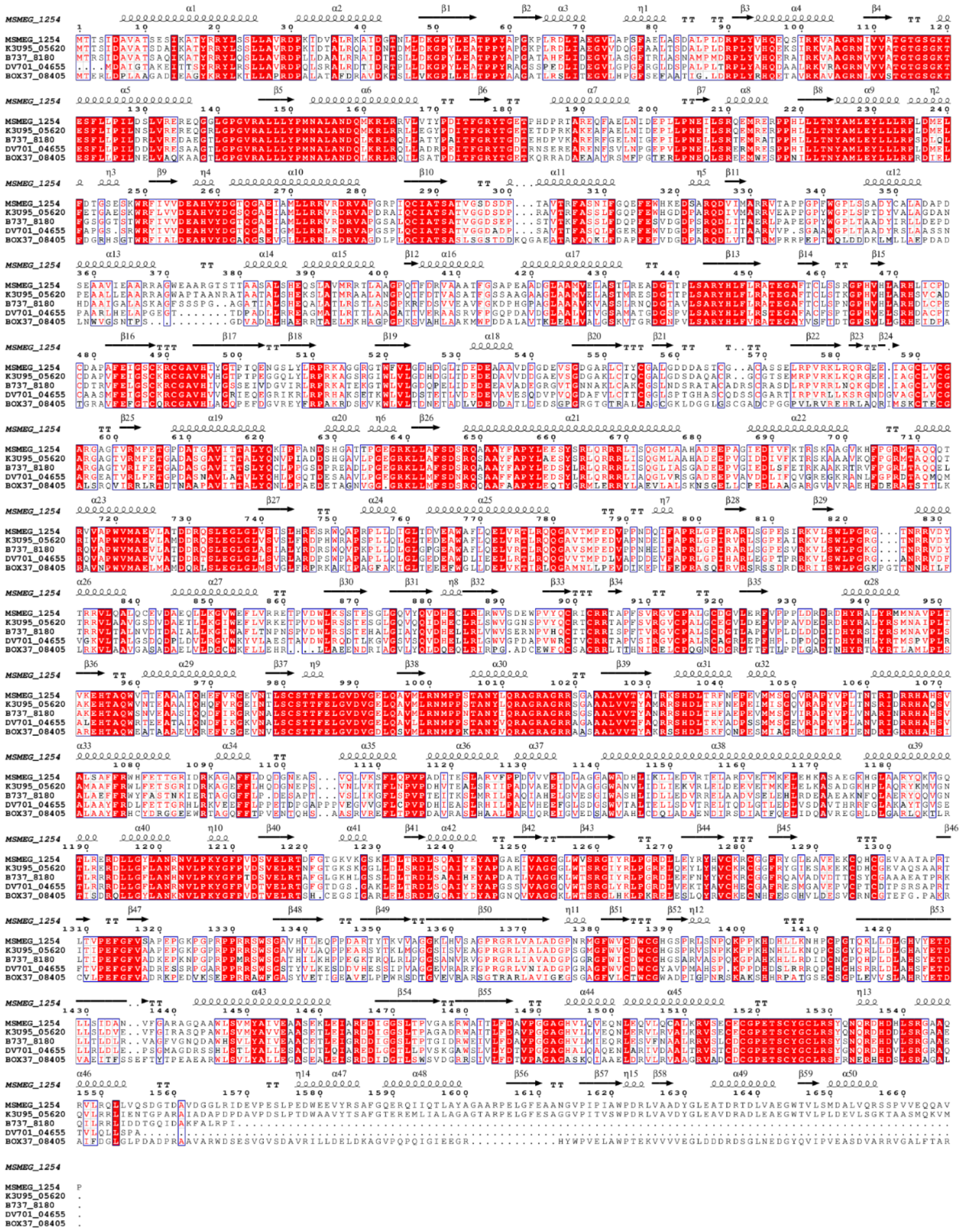

Fig. S2, related to Fig. 1. Amino acid sequence conservation analysis of DruE in the Type III Druantia system. The conservation of amino acids in DruE was analyzed and visualized using a color to represent the degree of conservation across homologous sequences.

A

Gene	Signature domain	Putative function
DurH	No domin	No
DurE	pfam00270	DEAD/DEAH box helicase
	pfam00271	Helicase conserved C-terminal domain
	pfam09369	DUF1998

B

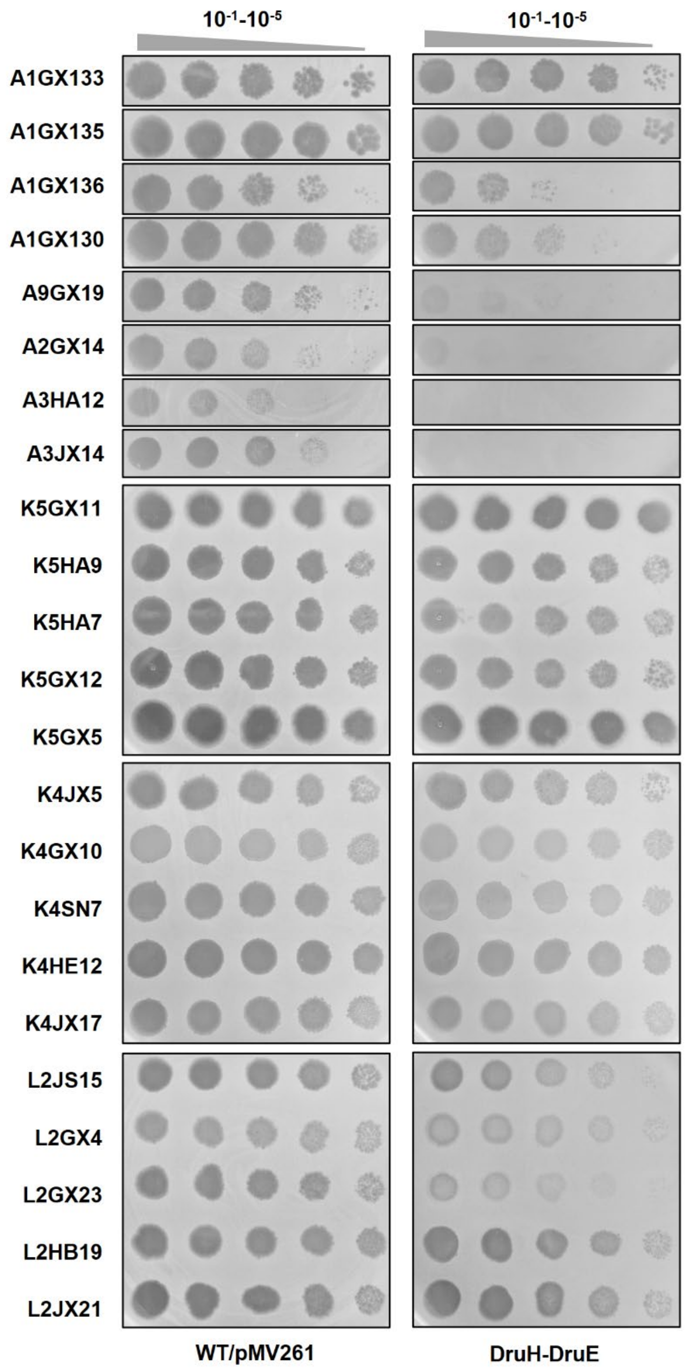

Fig. S3, related to Fig.1. Phage phenotypes assays for the DruH-DruE overexpression strain.

(A) Gene organization and domain annotation of the Type III Druantia system.The genetic architecture of the type III Druantia system, including its constituent genes and their associated functional domains, is depicted.
(B) Serial dilution assays for antiphage activity. Tenfold serial dilutions of a panel of genetically diverse mycobacteriophages were spotted onto lawns of *M. smegmatis* strains, including the wild-type (WT/pMV261) and the overexpression strain (DruH-DruE). The names of the tested phages are indicated on the left.

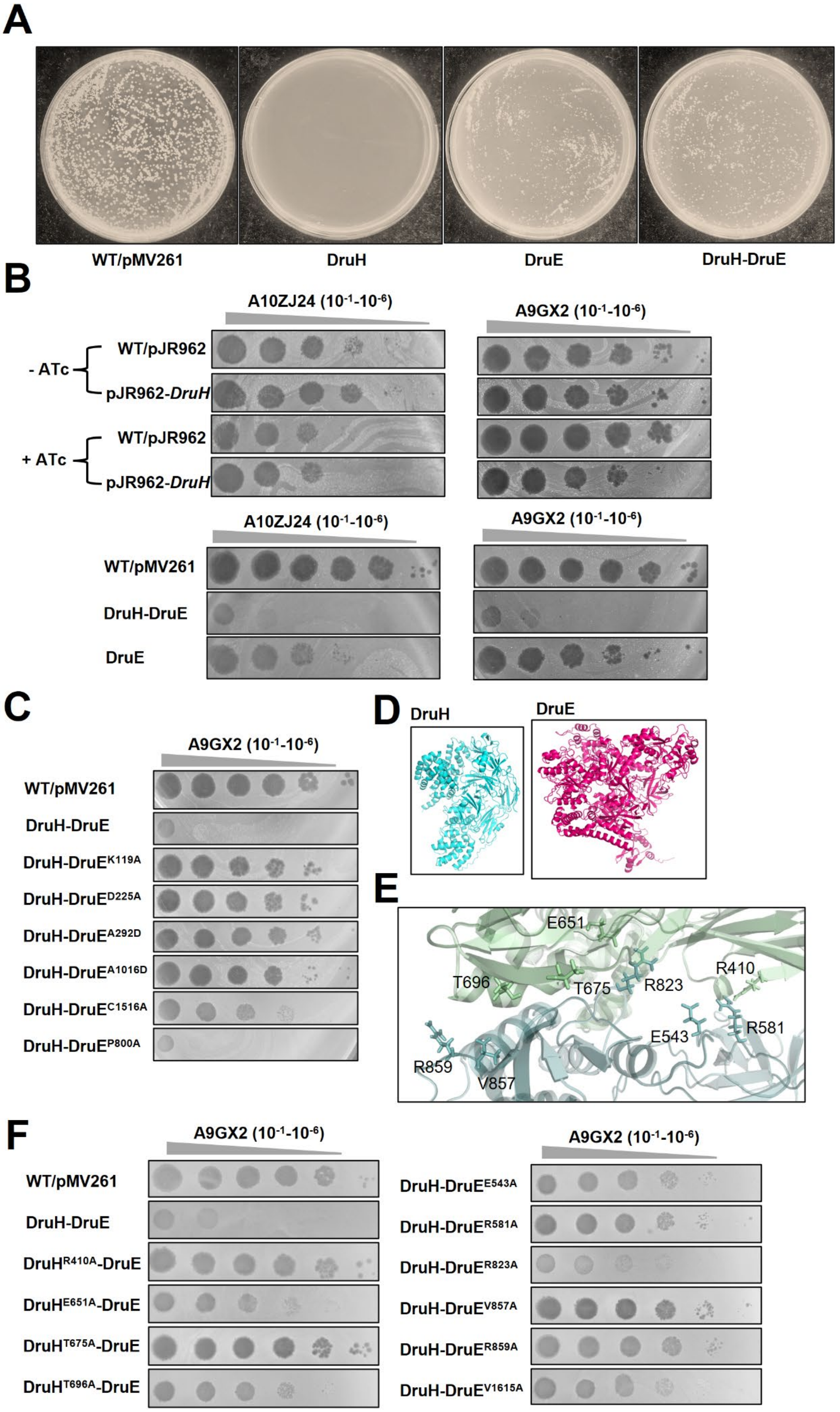
A
WT/pMV261
DruH
DruE
DruH-DruE
B
A10ZJ24 (10-1-10-6)
A9GX2 (10-1-10-6)
- ATc
WT/pJR962
pJR962-DruH
+ ATc
WT/pJR962
pJR962-DruH
A10ZJ24 (10-1-10-6)
A9GX2 (10-1-10-6)
WT/pMV261
DruH-DruE
DruE
C
A9GX2 (10-1-10-6)
WT/pMV261
DruH-DruE
DruH-DruEK119A
DruH-DruED225A
DruH-DruEA292D
DruH-DruEA1016D
DruH-DruEC1516A
DruH-DruEP800A
D
DruH
DruE
E
E651
T696
T675
R823
R410
R581
E543
R859
V857
F
A9GX2 (10-1-10-6)
WT/pMV261
DruH-DruE
DruHR410A-DruE
DruHE651A-DruE
DruHT675A-DruE
DruHT696A-DruE
A9GX2 (10-1-10-6)
DruH-DruEE543A
DruH-DruER581A
DruH-DruER823A
DruH-DruEV857A
DruH-DruER859A
DruH-DruEV1615A

Fig. S4, related to Fig. 2. Antiphage A9GX2 phenotype assays for type III Druantia system containing mutations in DruH or DruE.

(A) Growth of different *M. smegmatis* strains on 7H10 solid medium. Strains included the wild-type (WT/pMV261) and DruH, DruE, and DruH-DruE overexpression strains.
(B) Antiphage activity of the type III Druantia system. Tenfold serial dilution plaque assays were performed to evaluate the infection efficiency of phages A10ZJ24 and A9GX2 on *M. smegmatis* strains expressing the type III Druantia system or individual components (DruH or DruE)..
(C) Functional analysis of DruE mutations in the Druantia system. The phage resistance of the type III Druantia system was tested using strains harboring mutations in DruE, revealing the contribution of specific residues to antiphage activity.
(D) Structural prediction of DruH and DruE. The AlphaFold-predicted structures of DruH and DruE were generated to elucidate their molecular architecture and potential interaction interfaces.
(E) DruH-DruE complex interface analysis. The AlphaFold-predicted structure of the DruH-DruE complex was analyzed to identify key residues at the interaction interface, providing insights into the molecular basis of their functional partnership.
(F) Functional impact of DruH-DruE interface mutations. Mutations at the DruH-DruE interface were introduced based on residues with protein-protein contacts. These mutants were tested to determine their effects on the system's ability to confer phage resistance, highlighting the importance of specific interactions for antiphage activity.

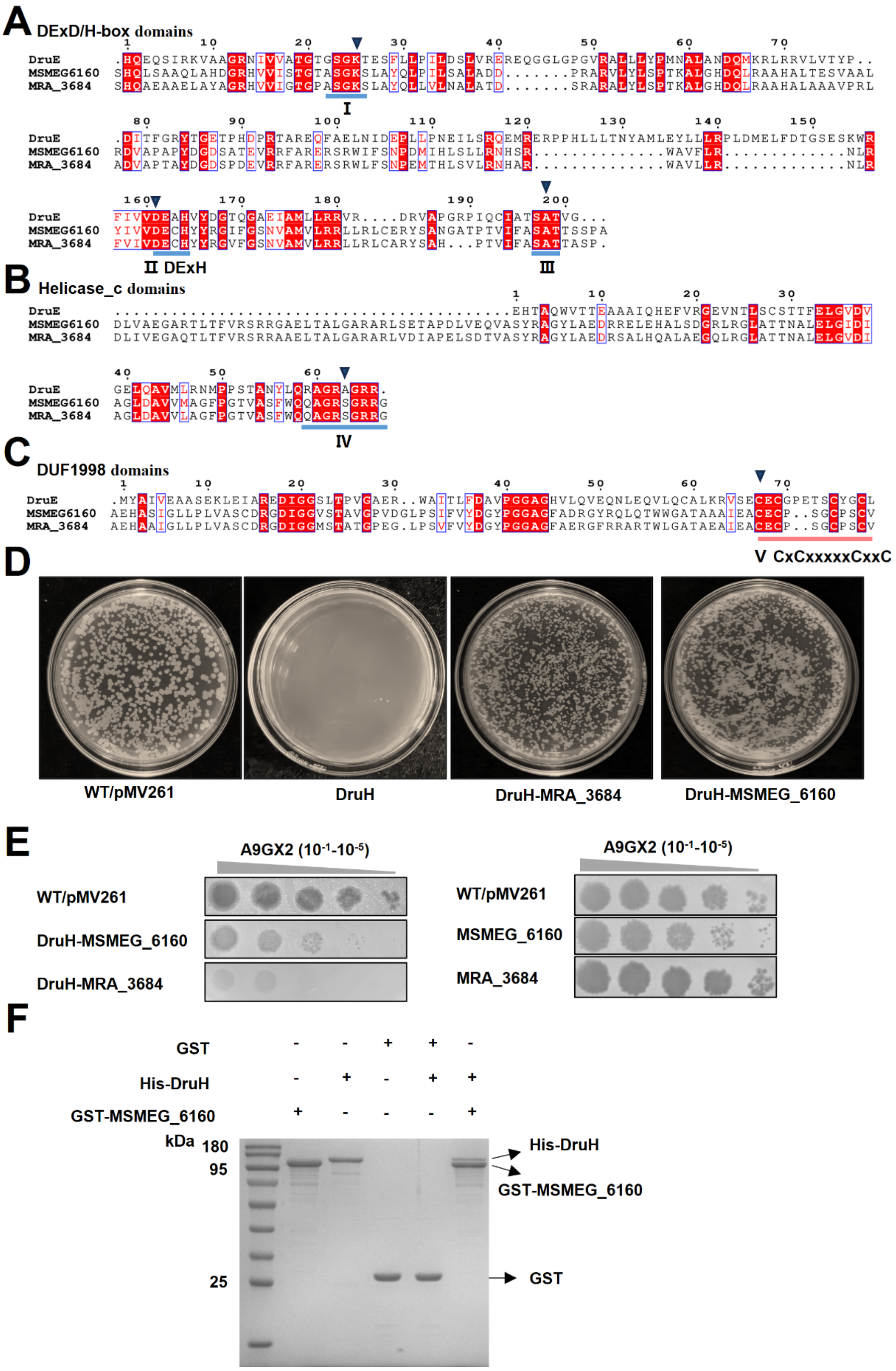

Fig. S5, related to Fig. 3. Antiphage activity of DruE homologous protein in *Mycobacterium*.

(A-C) Amino acid sequence conservation analysis of DExD/H-box, helicase_C and DUF1998 structural domains of DruE, MSMEG_6160, and MRA_3684Mra_3684. The superfamily II ATPase motifs I, II, III and IV are highlighted in blue, V represents tetracysteine cluster. The triangles represent DruE point-mutation sites.

(D) Growth of different *M. smegmatis* strains on 7H10 solid medium. WT/pMV261 indicates the wild-type strain. WT/pMV261 represents the wild-type strain. DruH, DruH-MRA_3684Mra_3684, DruH-MSMEG_6160 represent *M. smegmatis* strains with the corresponding gene overexpression, respectively.

(E) Phage plaque formation ability assays. 2 μL of serial dilutions of A9GX2 phage were spotted on lawns of mycobacterial cells (OD_{600} =1.0) and plaque formation was assessed after 24h of incubation at 37 °C.
(F) GST pull-down assays for the interaction between DruH and MSMEG_6160. The GST tag protein was used as a control.

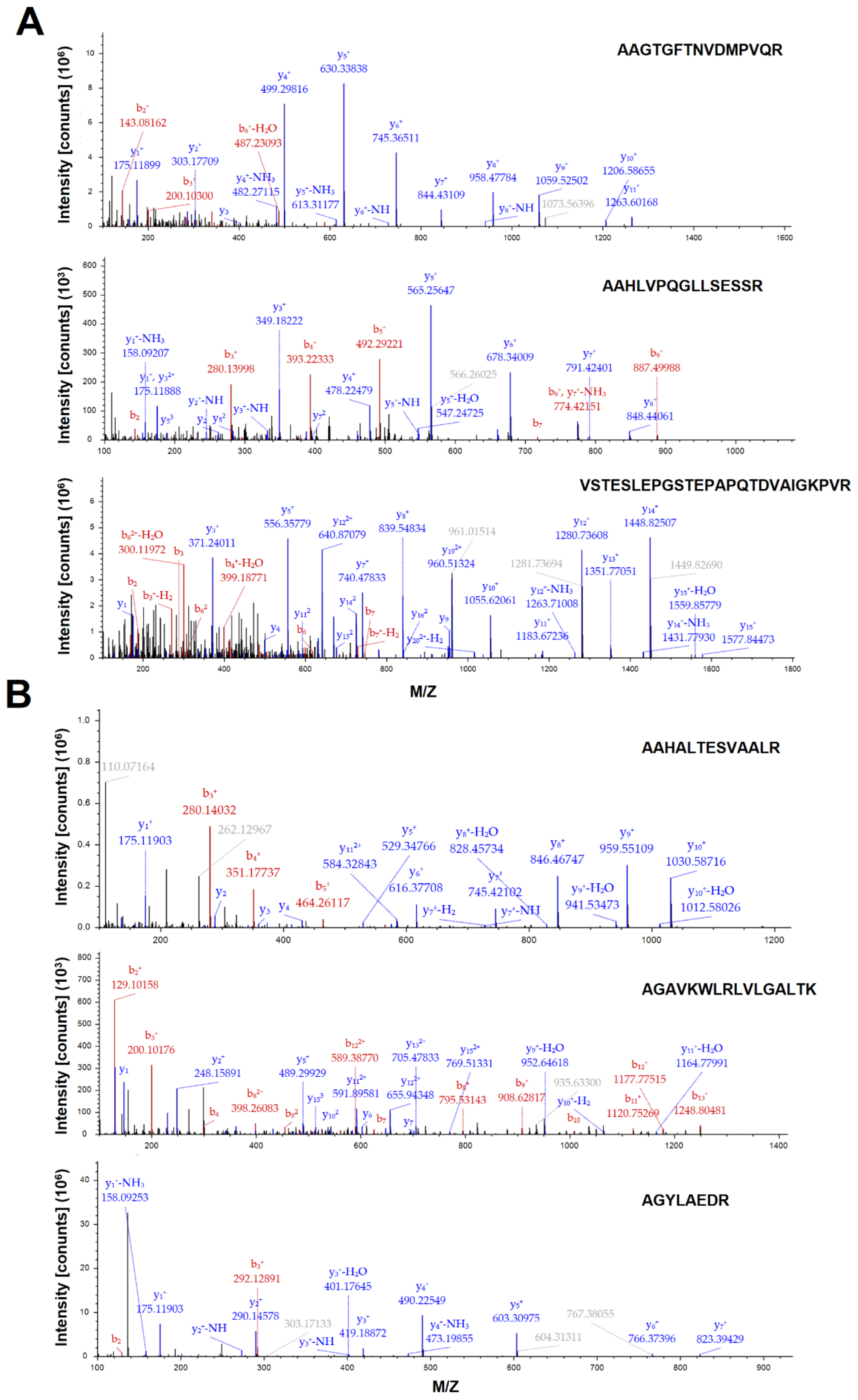

Fig. S6, related to Fig. 3. Identification of pull-down protein samples by nano-

liquid chromatography-mass spectrometry (NanoLC-MS).

(A) NanoLC-MS assays for specific peptide fragments of DruH. DruH-specifc peptide fragments with the amino acid sequences AAGTGFTNVDMPVQR, AAHLVPQGLLSESSR and VSTESLEPGSTEPAPQTDVAIGKPVR were identified.
(B) NanoLC-MS assays for specific peptide fragments of MSMEG_6160 protein. MSMEG_6160-specific peptide fragments with the amino acid sequences AAHALTESVAALR, AGAVKWLRLVLGALTK and AGYLAEDR were identified.

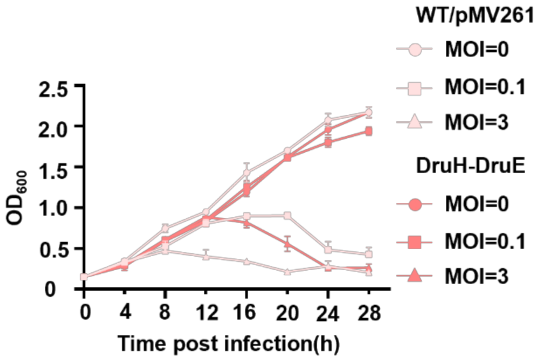

Fig. S7, related to Fig. 4. Growth curves for *M. smegmatis* cells harboring the DruH-DruE system or an empty vector, infected with the A10ZJ24 phage at a multiplicity of infection (MOI) of 0, 0.1, or 3.

Data points represent mean ± SEM of three biological replicates (n = 3).

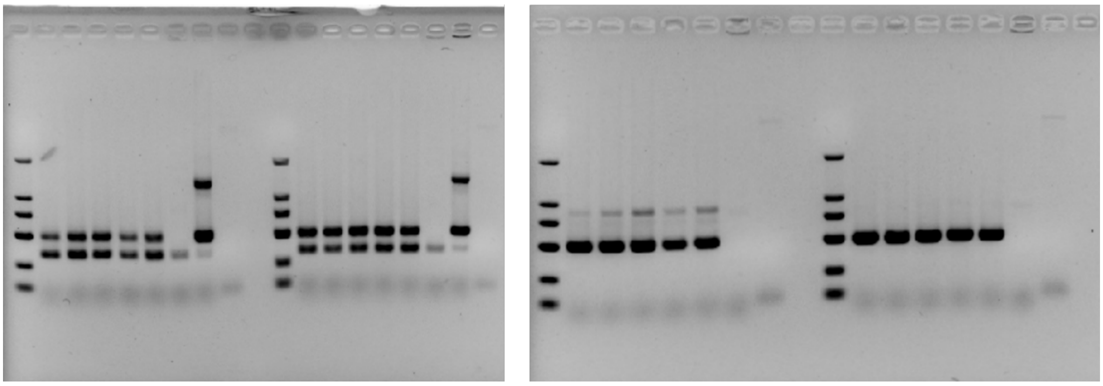

Fig. S8, related to Fig. 5. Phage DNA circularization is disrupted by the Type III Druantia system.

Uncropped gel Images, related to Fig. 5B.

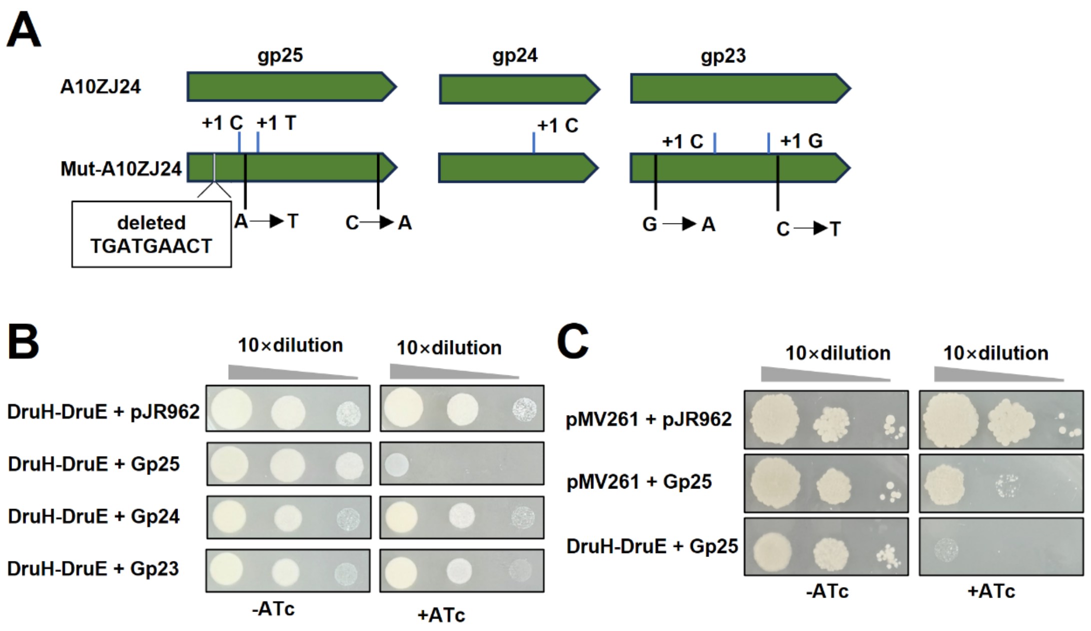

Fig. S9, related to Fig. 6. Analysis of the mutated genes that confer phage to escape the type III Druantia defense system

(A) Location of mutations found in phage that escape from the type III Druantia defense.

(B-C) Serial dilutions of cells were incubated in conditions with (+) or without (-) anhydrous tetracycline, bacterial viability was measured for strains in which the type III Druantia system was co-expressed with phage genes (*gp*23, *gp*24, *gp* 25).

pMV261+ pJR962 represents the wild-type strain. DruH-DruE + Gp25 represents the DruH-DruE strain with recombinant vector pJR962-*gp25*, in which Gp25 expression is induced by ATc. pMV261 + Gp25 represents the ATc-induced strain for Gp25 expression strain with an empty pMV261 vector. pMV261 + Gp25 represents the ATc-induced strain for gp25 expression strain with an empty pMV261 vector. DruH-DruE + Gp23 or 24 represents the DruH-DruE strain with recombinant vector pJR962-*gp23* or pJR962-*gp24*, in which Gp23 or Gp23 expression is induced by ATc.

Supplementary Table 1. PCR primers used in this study.

Primers name	Sequence 5'-3'	Usage
Ms1253-F	CGCGGTACCAGATCTTTAAATGTCTGCCCATTGC ATTAGCCGTTG	Cloned to pMV261
Ms1253-R	TCCAGCCCCCGATCCGACACTCAATACTGGTTGT CATTCCGCTTC	Cloned to pMV261
Ms1253pro-F	CGCGGTACCAGATCTTTAAATGTCTGCCCATTGC ATTAGCCGTTG	Cloned to pMV261
Ms1253pro-R	GCATCAATACTGGTTGTCATTCCTTTTCCGTCCTC GACCCTTCGT	Cloned to pMV261
Ms1254-F	GGGTCGAGGACGGAAAAGGAATGACAACCAGT ATTGATGCAGTTG	Cloned to pMV261
Ms1254-R	TTAACTACGTCGACATCGAT TCATGGAACAGCTTGCTGCTCGACC	Cloned to pMV261
Ms1253-54-F	CGCGGTACCAGATCTTTAAA TGTCTGCCCATTGCATTAGCCGTTGT	Cloned to pMV261
Ms1253-54-R	TTAACTACGTCGACATCGAT TCATGGAACAGCTTGCTGCTCGACC	Cloned to pMV261
1253-54(54K119A)-S1/F:	CGCGGTACCAGATCTTTAAATGTCTGCCCATTGC ATTAGCCGTTG	Cloned to pMV261
1253-54(54K119A)-S1/R:	GCCTGACCCAGTTCCGGTGGCGACCA	Cloned to pMV261
1253-54(54K119A)-S2/F:	CCACCGGAACTGGGTCAGGCGCAACCGAATCGT TCCTCCTACCGA	Cloned to pMV261
1253-54(54K119A)-S2/R:	CCGATGTTGCAATGCACTGGATGGGACGGCCAG GAGCCACTCGGT	Cloned to pMV261
1253-54(54K119A)-S3/F:	CCAGTGCATTGCAACATCGGCCACT	Cloned to pMV261
1253-54(54K119A)-S3/R:	TTAACTACGTCGACATCGATTCATGGAACAGCTT GCTGCTCGACC	Cloned to pMV261
1253-54(54D255A)-S1/F	CGCGGTACCAGATCTTTAAATGTCTGCCCATTGC ATTAGCCGTTG	Cloned to pMV261
1253-54(54D255A)-S1/R	GACGACGATGAATCGCCACTTGGAT	Cloned to pMV261
1253-54(54D255A)-S2/F	AGTGGCGATTCATCGTCGTCGCCGAAGCCCACGT CTACGACGGGA	Cloned to pMV261
1253-54(54D255A)-S2/R	CCACCATGGCTGCAAGTCCGTCTGCGGCTTCGGG TGCGCTGCCGA	Cloned to pMV261
1253-54(54D255A)-S3/F	CGGACTTGCAGCCATGGTGGAGCTC	Cloned to pMV261
1253-54(54D255A)-S3/R	TTAACTACGTCGACATCGATTCATGGAACAGCTT GCTGCTCGACC	Cloned to pMV261
1253-54(54A292D)-S1/F	CGCGGTACCAGATCTTTAAATGTCTGCCCATTGC ATTAGCCGTTG	Cloned to pMV261
1253-54(54A292D)-S1/R	CGATGTTGCAATGCACTGGATGGGA	Cloned to pMV261

1253-54(54A292D)-S2/F	TCCAGTGCATTGCAACATCGGACACTGTCGGAAGTGATTCGGATC	Cloned to pMV261
1253-54(54A292D)-S2/R	GCCCCCTCGACGACAGGCAGGTGAATGCACCCTCGGTTGCCCTGA	Cloned to pMV261
1253-54(54A292D)-S3/F	CTGCCTGTCGTCGAGGGGGCCACAT	Cloned to pMV261
1253-54(54A292D)-S3/R	TTAACTACGTCGACATCGATTCATGGAACAGCTTGCTGCTCGACC	Cloned to pMV261
1253-54(54A1016D)-S1/F	CGCGGTACCAGATCTTTAAATGTCTGCCCATTGCATTAGCCGTTG	Cloned to pMV261
1253-54(54A1016D)-S1/R	ACGACCGGCACGCTGAAGGTAGTTG	Cloned to pMV261
1253-54(54A1016D)-S2/F	ACCTTCAGCGTGCCGGTCGTGATGGGCGCCGGTCAGGAGCCGCCG	Cloned to pMV261
1253-54(54A1016D)-S2/R	TCTGCCCAACCTTTTGATATCGCGCAGCGAGACCGTGTTTACCCT	Cloned to pMV261
1253-54(54A1016D)-S3/F	ATATCAAAAGGTTGGGCAGACACTG	Cloned to pMV261
1253-54(54A1016D)-S3/R	TTAACTACGTCGACATCGATTCATGGAACAGCTTGCTGCTCGACC	Cloned to pMV261
1253-54(54C1516A)-S1/F	CGCGGTACCAGATCTTTAAATGTCTGCCCATTGCATTAGCCGTTG	Cloned to pMV261
1253-54(54C1516A)-S1/R	CTCACTCACCCGTTTGAGAGCGCAT	Cloned to pMV261
1253-54(54C1516A)-S2/F	CTCTCAAACGGGTGAGTGAGGCTGAGTGCGGGCCGGAAACGTCGT	Cloned to pMV261
1253-54(54C1516A)-S2/R	TTAACTACGTCGACATCGATTCATGGAACAGCTTGCTGCTCGACC	Cloned to pMV261
(53R536A)-S1/F	CGCGGTACCAGATCTTTAAATGTCTGCCCATTGCATTAGCCGTTG	Cloned to pMV261
(53R536A)-S1/R	caaattggagtaaaggtcgccgCaagcccctcgactacagtgaat	Cloned to pMV261
(53R536A)-S2/F	gcgacctttactccaatttggcgacgt	Cloned to pMV261
(53R536A)-S2/R	TTAACTACGTCGACATCGATTCATGGAACAGCTTGCTGCTCGACC	Cloned to pMV261
(53D240A)-S1/R	ctccatcctgatgccaccgcgGcgagcagtGcaaacgaacgatcggccacgtcg	Cloned to pMV261
(53D240A)-S2/F	gcggtggcatcaggatggagcgtcg	Cloned to pMV261
(53E842A)-S1/R	gccctaacggtccagagccgtGcgaaatcaatcacctcgacaggtt	Cloned to pMV261
(53E842A)-S2/F	cggctctggaccgttagggcgctgct	Cloned to pMV261
(53R864/865A)-S1/R	tagacctcagtatcgatagccGctGcgactGcccgaatgcgctgtccaagact	Cloned to pMV261
(53R864/865A)-S2/F	gctatcgatactgaggtctacgcaaatcc	Cloned to pMV261
(53E1096/1099A)-S1/R	gtgaccaactgaggaacaaccGcagccaacGccgcccaggctcgtttctctcga	Cloned to pMV261

(53E1096/1099A)-S2/F	gttgttcctcagttggtcaccatcgac	Cloned to pMV261
(53D1108A)-S1/R	atcagctcggcgataatcaagGcgatggtgaccaactgaggaacaa	Cloned to pMV261
(53D1108A)-S2/F	ttgattatcgccgagctgatcatcgg	Cloned to pMV261
(53E998A)-S1/R	accgcgaggatgcgggacgacGcggaaagcaagccttgaggcacaa	Cloned to pMV261
(53E998A)-S2/F	tcgtcccgcatcctcgcggtgat	Cloned to pMV261
(53 R410A)-S1/R	ATCTGGAACGATCCGCTGACGGCCAGCGCGTCG CG	Cloned to pMV261
(53 R410A)-S2/F	gtcagcggatcgttccagatcatcg	Cloned to pMV261
(53 E651A) -S1/R	ACACCCACGTCGAGGACAAGTGCTCCAGGCCGC TC	Cloned to pMV261
(53 E651A) -S2A/F	cttgtcctcgacgtgggtgtggtag	Cloned to pMV261
(53 T675A) -S1/R	CGCCCGCTCTTCGGCGAAAGGGCCTGAATCGAC CG	Cloned to pMV261
(53 T675A) -S2/F	ctttcgccgaagagcgggcgtatcg	Cloned to pMV261
(53 T696A) -S1/R	ACGTGCGGATACTCACCCAAAGCGTCTGCAATCT C	Cloned to pMV261
(53 T696A) -S2/F	ttgggtgagtatccgcacgtcagcc	Cloned to pMV261
(54 E543A) -S1/R	GTCACCGTCGTCGACGGCGGCGGCT	Cloned to pMV261
(54 E543A) -S2/F	ccgccgtcgacgacggtgac gCagtctctggcgat	Cloned to pMV261
(54 R581A) -S1/R	GAGTTTGCGCACTGGGCGGAGCTCG	Cloned to pMV261
(54 R581A) -S2/F	tccgcccagtgcgcaaactc GCgcagcgtggcgag	Cloned to pMV261
(54 R823A) -S1/R	GCCCGGCAGCCATGAGAGGACCTTT	Cloned to pMV261
(54 R823A) -S2/F	tcctctcatggctgccgggc GCtggaacgaatcgc	Cloned to pMV261
(54 V857A) -S1/R	AAGGAACTCCCATACGCCCTTCAAG	Cloned to pMV261
(54 V857A) -S2/F	agggcgtatgggagttcctt gCgcgcagagaaact	Cloned to pMV261
(54 R859A) -S1/R	GCGCACAAGGAACTCCCATACGCCC	Cloned to pMV261
(54 R859A) -S2/F	tatgggagttccttgtgcgc GCagaaactccggtg	Cloned to pMV261
(54 V1615A) -S1/R	TCCGTTGGCGGCCTCGAATCCGAGT	Cloned to pMV261
(54 V1615A) -S2/F	gattcgaggccgccaacgga gCtcctatccctatt	Cloned to pMV261
Lysogen-F	CGTGATAGTCCAGACCGGCA	PCR
Lysogen-R	TTCGTGTCCCCCTGGACCCA	PCR
bacterial-F	ATGGCAAAGAAAGTGACCGT	PCR
bacterial-R	CTAAGTTGCCGCGTGGAATG	PCR
Phage-F	ATGGAAGACGACGGGACCAA	PCR
Phage-R	CTACGGCTCCACGGGTGGGA	PCR
Circular-F	CAGAACGGGCTGCCGGTAGT	PCR
Circular-R	GCGATCCGCTGTTGAGTTGT	PCR
pTRG-Ms1253-F	GCGGCCGC GTGTCGTTATGTCCCGGTTTACCGC	Cloned to pTRG

pTRG-Ms1253-R	TCTAGA TCAATACTGGTTGTCATTCCGCTTC	Cloned to pTRG
(53D410A)-S1/R	ATCTGGAACGATCCGCTGACGGCCAGCGCGTCG CG	Cloned to pTRG
(53D410A)-S2/F	gtcagcggatcgttccagatcatcg	Cloned to pTRG
(53 E651A) -S1/R	ACACCCACGTCGAGGACAAGTGCTCCAGGCCGC TC	Cloned to pTRG
(53 E651A) -S2A/F	cttgtcctcgacgtgggtgtggtag	Cloned to pTRG
pBT-Ms1254-F	GGCCTGAAGAGACGTTTGGCATGACAACCAGTA TTGATGCAGTTG	Cloned to pBT
pBT-Ms1254-R	ACGCACGCCCGAGATGATACTCATGGAACAGCT TGCTGCTCGACC	Cloned to pBT
(54 R859A) -S1/R	GCGCACAAGGAACTCCCATACGCCC	Cloned to pBT
(54 R859A) -S2/F	tatgggagttccttgtgcgc GCagaaactccggtg	Cloned to pBT
(54 R823A) -S1/R	GCCCGGCAGCCATGAGAGGACCTTT	Cloned to pBT
(54 R823A) -S2/F	tcctctcatggctgccgggc GCtggaacgaatcgc	Cloned to pBT
COS-F	CTCTGGTATCGCGGATAGGG	qRT-PCR
COS-R	GGGTATCGCTGCAGGTAGG	qRT-PCR
16SrRNART-F	GATACGGGCAGACTAGAGTA	qRT-PCR
16SrRNART-R	GGGTATCTAATCCTGTTCGC	qRT-PCR
Dut-Ms1253-F:	ACCATCATCACCACAGCCAGGTGTCGTTATGTCC CGGTTTACCGC	Cloned to pRSF-Dut
Dut-Ms1253-R:	CTTAAGCATTATGCGGCCGCTCAATACTGGTTGT CATTCCGCTTC	Cloned to pRSF-Dut
Gp48-F:	TCAGTGATAGAGAAGGCGGTATGAGTACCAGCA CCATCACGACGC	Cloned to pLJR-962
Gp48-R：	GCCTTCTGCTTAGCTAATCATTAGAGGAGTCCGA TCTGACCCATG	Cloned to pLJR-962
Gp49-F:	TCAGTGATAGAGAAGGCGGTGTGGACACCCTCG TACAGCTTGAGG	Cloned to pLJR-962
Gp49-R：	GCCTTCTGCTTAGCTAATCATCATTCGAGCCCCC AGGGCCTCGTC	Cloned to pLJR-962
Gp50-F:	TCAGTGATAGAGAAGGCGGTATGAAGGGCTTCC GGGAGGAGATCG	Cloned to pLJR-962
Gp50-R：	GCCTTCTGCTTAGCTAATCATTACCTCCTATTGA ATTGCAACGCC	Cloned to pLJR-962
Mra_3684-F:	CGCGGTACCAGATCTTTAAAATACATCCGCGGA ACCGTCTTCGGG	Cloned to pMV261
Mra_3684-R:	TTAACTACGTCGACATCGATTCACGGTGATTCCT CACTTAACTCG	Cloned to pMV261
Ms6160-F:	CGCGGTACCAGATCTTTAAAAGCCGAAGCCCTTC TCCGCGTTGAA	Cloned to pMV261

Ms6160-R:	TTAACTACGTCGACATCGATCTACTTCGTCAGCG CGCCGAGCACC	Cloned to pMV261
53-Mra_3684-F1:	CGCGGTACCAGATCTTTAAATGTCTGCCCATTGC ATTAGCCGTTG	Cloned to pMV261
53-Mra_3684-F1:	TGGCTGCCGAAACTCGCCATTCAATACTGGTTGT CATTCCGCTTC	Cloned to pMV261
53-Mra_3684-F2:	GGAATGACAACCAGTATTGAATGGCGAGTTTCG GCAGCCACCTGC	Cloned to pMV261
53-Mra_3684-R2:	TTAACTACGTCGACATCGATTCACGGTGATTCCT CACTTAACTCG	Cloned to pMV261
53-Ms6160-F1:	CGCGGTACCAGATCTTTAAATGTCTGCCCATTGC ATTAGCCGTTG	Cloned to pMV261
53- Ms6160-F1:	TCCAGCCCCCGATCCGACACTCAATACTGGTTGT CATTCCGCTTC	Cloned to pMV261
53- Ms6160-F2:	GGAATGACAACCAGTATTGAGTGTCGGATCGGG GGCTGGAATTCG	Cloned to pMV261
53- Ms6160-R2:	TTAACTACGTCGACATCGATCTACTTCGTCAGCG CGCCGAGCACC	Cloned to pMV261

Supplementary Table 2. Strains, phages and plasmids used in this study.

**Plasmid, phage or Strain**	**Relevant genotype or feature**	**Source or reference**
Plasmid		
pRSF-Dut	*Kan*^{r}, T7 lac promoter, N-terminal His_6	Novagen
pRSF -*MSMEG_5860*	*MSMEG_5860* inserted in *BamH* I-*Hind* Ⅲ of pRSF-Dut	This study
pJR962	including Sth1 sgRNA scaffold and dCas9, *Hyg*^{r} instead of *Kan*^{r})This study	Previous study [S1]
pJR962-*A10ZJ24gp48*	*A10ZJ24gp48* inserted in *Cla* I-*Not* Iof pLJR962	This study
pJR962- *A10ZJ24gp49*	*A10ZJ24gp49* inserted in *Cla* I-*Not* I of pLJR962	This study
pJR962- *A10ZJ24gp50*	*A10ZJ24gp50* inserted in *Cla* I-*Not* I of pLJR962	This study
pJR962-*Mut-gp48*	*Mut-gp48* inserted in *Cla* I-*Not* I of pLJR962	This study
pJR962-*MSMEG_1253*	*MSMEG_1253* inserted in *Cla* I-*Not* I of pLJR962	This study
pMV261	*Kan*^{r}, pAL5000 replicon	Previous study[S1]
pMV261-*MSMEG_1253*-1254	*MSMEG_1253*-1254 inserted in *Xba* I-*Hind* III sites of pMV261	This study
pMV261-*MSMEG_1253*	*MSMEG_1253* inserted in *Xba* I-*Hind* III sites of pMV261	This study
pMV261-*MSMEG_1254*	*MSMEG_1254* inserted in *Xba* I-*Hind* III sites of pMV261	This study
pMV261-*MSMEG_6160*	*MSMEG_6160* inserted *in Xba* I-*Hind* III *of* pMV261	This study
pMV261-*Mra_3684*	*Mra_3684 inserted in Xba* I-*Hind* III *of* pMV261	This study
pMV261-*DruH/MSMEG_6160*	*MSMEG_1253/MSMEG_6160 inserted in Xba* I-*Hind* III *of* pMV261	This study
pMV261-*DruH/Mra_3684*	*MSMEG_1253/Mra_3684 inserted in Xba* I-*Hind* III *of* pMV261	This study
pBT	Bacterial 2-hybrid assay bait vector	Stratagene
pTRG	Bacterial 2-hybrid assay bait vector	Stratagene
pBT-*MSMEG_1254*	*MSMEG_1254*in *Not* I-*Xba* I sites of pBT	This study
pTRG-*MSMEG_1253*	*MSMEG_1253* in *Not* I-*Xba* I sites of pTRG	This study
Strain		
E. coli BL21 (DE3)		TaKaRa
M. smegmatis MC^2 155		ATCC
M. tuberculosis H37Ra		ATCC
WT/pMV261	*M. smegmatis* MC^2 155 with pMV261	This study
DruH-DruE	*M. smegmatis* MC^2 155 with pMV261-*MSMEG_1253-1254*	This study
DruE	*M. smegmatis* MC^2 155 with pMV261-*MSMEG_1254*	This study
DruH	*M. smegmatis* MC^2 155 with pMV261-*MSMEG_1253*	This study
$DruH^{E1096/1099A}$-DruE	*M. smegmatis* MC^2 155 with pMV261-*MSMEG_1253(*E1096/1099A*)-1254*	This study
$DruH^{E842A}$-DruE	*M. smegmatis* MC^2 155 with pMV261-*MSMEG_1253*(E842A)-*1254*	This study
$DruH^{R536A}$-DruE	*M. smegmatis* MC^2 155 with pMV261-*MSMEG_1253*(R536A)-*1254*	
$DruH^{D240A}$-DruE	*M. smegmatis* MC^2 155 with pMV261-*MSMEG_1253*(D240A)-*1254*	

**Continued**		
**Plasmid, phage or Strain**	**Relevant genotype or feature**	**Source or reference**
DruH$^{R864/865A}$-DruE	*M. smegmatis* MC^2 155 with pMV261-*MSMEG_1253*(R864/865A)-*1254*	This study
DruH^{D1108A}-DruE	*M. smegmatis* MC^2 155 with pMV261-*MSMEG_1253*(D1108A)-*1254*	This study
DruH^{E998A}-DruE	*M. smegmatis* MC^2 155 with pMV261-*MSMEG_1253*(E998A)-*1254*	This study
DruH-DruE^{K119A}	*M. smegmatis* MC^2 155 with pMV261-*MSMEG_1253-1254*(K119A)	This study
DruH-DruE^{D225A}	*M. smegmatis* MC^2 155 with pMV261-*MSMEG_1253-1254*(D225A)	This study
DruH-DruE^{A292D}	*M. smegmatis* MC^2 155 with pMV261-*MSMEG_1253-1254*(A292D)	This study
DruH-DruE^{A1016D}	*M. smegmatis* MC^2 155 with pMV261-*MSMEG_1253-1254*(A1016D)	This study
DruH-DruE^{C1516A}	*M. smegmatis* MC^2 155 with pMV261-*MSMEG_1253-1254*(C1516A)	This study
DruH-DruE^{P800A}	*M. smegmatis* MC^2 155 with pMV261-*MSMEG_1253-1254*(P800A)	This study
DruH^{R410A}-DruE	*M. smegmatis* MC^2 155 with pMV261-*MSMEG_1253* (R410A) -*1254*	This study
DruH^{E651A}-DruE	*M. smegmatis* MC^2 155 with pMV261-*MSMEG_1253* (E651A) -*1254*	This study
DruH^{T675A}-DruE	*M. smegmatis* MC^2 155 with pMV261-*MSMEG_1253* (T675A) -*1254*	This study
DruH^{T696A}-DruE	*M. smegmatis* MC^2 155 with pMV261-*MSMEG_1253* (T696A) -*1254*	This study
DruH-DruE^{E543A}	*M. smegmatis* MC^2 155 with pMV261-*MSMEG_1253-1254*(E543A)	This study
DruH-DruE^{R581A}	*M. smegmatis* MC^2 155 with pMV261-*MSMEG_1253-1254*(R581A)	This study
DruH-DruE^{R823A}	*M. smegmatis* MC^2 155 with pMV261-*MSMEG_1253-1254*(R823A)	This study
DruH-DruE^{V857A}	*M. smegmatis* MC^2 155 with pMV261-*MSMEG_1253-1254*(V857A)	This study
DruH-DruE^{R859A}	*M. smegmatis* MC^2 155 with pMV261-*MSMEG_1253-1254*(R859A)	This study
DruH-DruE V1615A	*M. smegmatis* MC^2 155 with pMV261-*MSMEG_1253-1254*(V1615A)	This study
pMV261 + Gp25	*M. smegmatis* MC^2 155 with pMV261 and pJR962-*A10ZJ24gp25*	This study

Continued

**Plasmid, phage or Strain**	**Relevant genotype or feature**	**Source or reference**
DruH-DruE + Gp25	*M. smegmatis* MC^2 155 with pMV261-*MSMEG_1253-1254* and pJR962-*A10ZJ24gp25*	This study
DruH-DruE^{EA292D} + Gp25	*M. smegmatis* MC^2 155 with pMV261-*MSMEG_1253-1254* (A292D) and pJR962-*A10ZJ24gp25*	This study
DruH-DruE^{A1016D} + Gp25	*M. smegmatis* MC^2 155 with pMV261-*MSMEG_1253-1254* (A1016D) and pJR962-A10ZJ24*gp25*	This study
pMV261 + Mut-Gp25	*M. smegmatis* MC^2 155 with pMV261 and pJR962-Mut-*gp25*	This study
DruH-DruE + Mut-Gp25	*M. smegmatis* MC^2 155 with pMV261-*MSMEG_1253-1254* and pJR962-Mut-*gp25*	This study
Mra/pMV261	*M. tuberculosis* H37Ra with pMV261	This study
(Mra_3684)	*M. tuberculosis* H37Rv with pMV261- *Mra_3684*	This study
(DruH-Mra_3684)	*M. tuberculosis* H37Rv with pMV261-DruH/*Mra_3684*	This study
(DruH-DruE)	*M. tuberculosis* H37Rv with pMV261-*MSMEG_1253-1254*	This study
(DruH-DruE^{K119A})	*M. tuberculosis* H37Rv with pMV261- *MSMEG_1253-1254* (K119A)	This study
(DruH-DruE^{D225A})	*M. tuberculosis* H37Rv with pMV261- *MSMEG_1253-1254* (D225A)	This study
(DruH-DruE^{A292D})	*M. tuberculosis* H37Ra with pMV261- *MSMEG_1253-1254* (A292D)	This study
(DruH-DruE^{A1016D})	*M. tuberculosis* H37Ra with pMV261- *MSMEG_1253-1254* (A1016D)	This study
DruH/DruE	E. coli reporter strain with pBT-*MSMEG_1254* and pTRG-*MSMEG_1253*	This study
DruH/DruE^{R823A}	E. coli reporter strain with pBT-*MSMEG_1254*(R823A) and pTRG-*MSMEG_1253*	This study
DruH/DruE^{R859A}	E. coli reporter strain with pBT-*MSMEG_1254*(R859A) and pTRG-*MSMEG_1253*	This study
DruH^{R410A}/DruE^{R823A}	E. coli reporter strain with pBT-*MSMEG_1254*(R823A) and pTRG-*MSMEG_1253*(R410A)	This study
DruH^{E651A}/DruE^{R823A}	E. coli reporter strain with pBT-*MSMEG_1254*(R823A) and pTRG-*MSMEG_1253*(E651A)	This study
A1ZJ29		This study
F1GX13		This study
A10ZJ24		This study
A22GX2		This study
A9GX2		This study
A1GX133		This study

Plasmid, phage or Phage	Relevant genotype or feature	Source or reference
A1GX133		This study
A1GX135		This study
A1GX136		This study
A1GX130		This study
A9GX19		This study
A2GX14		This study
A3HA12		This study
A3JX14		This study
K5GX11		This study
K5HA9		This study
K5HA7		This study
K5GX12		This study
K5GX5		This study
K4JX5		This study
K4GX10		This study
K4SN7		This study
K4HE12		This study
K4JX17		This study
L2JS15		This study
L2GX4		This study
L2GX23		This study
L2HB19		This study
L2JX21		This study
A1SD115		This study

References

Li, X., Long, X., Chen, L., Guo, X., Lu, L., Hu, L., & He, Z.-G. (2023). Mycobacterial phage TM4 requires a eukaryotic-like Ser/Thr protein kinase to silence and escape anti-phage immunity. Cell Host & Microbe, 31(9), 1469-1480.e4. https://doi.org/10.1016/j.chom.2023.07.005
